# Magnetic properties of iron cluster/chromium matrix nanocomposites

**DOI:** 10.3762/bjnano.6.117

**Published:** 2015-05-13

**Authors:** Arne Fischer, Robert Kruk, Di Wang, Horst Hahn

**Affiliations:** 1 Institute of Nanotechnology, Karlsruhe Institute of Technology (KIT), 76344 Eggenstein-Leopoldshafen, Germany; 2 KIT-TUD Joint Research Laboratory Nanomaterials, Technische Universität Darmstadt (TUD), 64287 Darmstadt, Germany

**Keywords:** cluster, cluster deposition, exchange bias, matrix

## Abstract

A custom-designed apparatus was used for the fine-tuned co-deposition of preformed Fe clusters into antiferromagnetic Cr matrices. Three series of samples with precisely defined cluster sizes, with accuracy to a few atoms, and controlled concentrations were fabricated, followed by a complete characterization of structure and magnetic performance. Relevant magnetic characteristics, reflecting the ferromagnetic/antiferromagnetic coupling between Fe clusters and the Cr matrix, i.e., blocking temperature, coercivity field, and exchange bias were measured and their dependence on cluster size and cluster concentration in the matrix was analyzed. It is evident that the blocking temperatures are clearly affected by both the cluster size and their concentration in the Cr matrix. In contrast the coercivity shows hardly any dependence on size or inter-cluster distance. The exchange bias was found to be strongly sensitive to the cluster size but not to the inter-cluster distances. Therefore, it was concluded to be an effect that is purely localized at the interfaces.

## Introduction

Today’s metallic alloys are prepared by using complex thermo-mechanical treatment steps, i.e., quenching, annealing combined with plastic deformation, in order to obtain the multicomponent multiphase structures optimized for advanced structural and functional applications [1]. Besides the pathways used during the preparation of the alloys, their final nano- and microstructure is determined strongly by the phase diagram limiting the extent of deviation from the well-defined thermodynamic equilibrium, which, for example, determines the volume fraction of precipitates or second phase particles and the composition of the matrix phase. Oxide dispersion strengthened alloys (ODS) are exceptions, as the distribution of oxide particles in the metallic matrix can be modified without the above mentioned constraints as the processing is done by mechanical alloying, not via the melt route followed by thermo-mechanical treatments. In metallic multiphase alloys, however, the ranges of precipitate sizes and the width of their distributions, as well as the chemical compositions of the precipitates and the matrix are severely limited by the thermodynamics of the alloy systems. Therefore, in any case the potential of alloy design will remain limited as long as thermo-mechanical treatment is employed for processing of alloys.

Simultaneous deposition of preformed clusters with pre-selected sizes ranging from a few atoms to thousands of atoms and of an atomic beam of another element onto a substrate opens a way to overcome this dilemma. There is a rich literature on the synthesis of charged clusters of basically any element and many alloy systems and their transfer into an ultra-high vacuum system (UHV). The deposition of the charged clusters onto substrates can be performed with variable impact energies. Such a process opens a new way for the synthesis of cluster-based alloys, i.e., multiphase alloys with extreme control of the fraction of clusters inside a matrix consisting of another element or alloy system. For the alloy system Fe/Ag it has been shown that full control over the overall composition of the two immiscible elements can be achieved [2]. One of the scopes of the experiments with Fe/Ag was to study the characteristics of the embedded Fe clusters. Since Ag is diamagnetic no noteworthy magnetic interaction takes place between matrix and the ferromagnetic clusters and it was possible to gain information about, e.g., the size of the embedded clusters via magnetic measurements. The intention of the present work is to go one step further to a more complex cluster/matrix system and to substitute the passive Ag matrix with a functional one, e.g., antiferromagnetic (AFM) Cr, leading to additional effects: At the interface between the ferromagnetic (FM) and the antiferromagnetic (AFM) phases a spin exchange coupling occurs and a part of the magnetic moments of the FM phase become pinned. This results in an increased magnetic anisotropy manifesting itself as an exchange bias effect (EB) [3]. The EB appears as a horizontal shift of the magnetization loops, the EB field *H*_eb_, and is usually accompanied by an increase of coercivity (*H*_c_) and of the blocking temperature (*T*_B_). The EB was first described by Meiklejohn and Bean in 1956 [4]. They investigated clusters with a FM cobalt core and an AFM cobalt oxide (CoO) shell and consequently observed the characteristic horizontal shift of the hysteresis loops recorded after field cooling the samples from temperatures above the Néel temperature (*T*_N_) of CoO.

Since its discovery the EB has been observed in numerous FM/AFM combinations such as core/shell clusters [5–6], thin film systems [7–8] and also cluster/matrix combinations [9–11]. So far, most of the research has been focused on thin film systems due to their commercial importance for reading heads in magnetic data storage [12]. Since many difficulties arise in fabricating FM cluster/AFM matrix systems in a strictly controlled way there are fewer studies compared to thin films. In principle there are two main approaches to the fabrication of FM cluster/AFM matrix systems. The first is to co-evaporate several materials or to chemically produce a compound in a first step and to induce the formation of FM precipitates in a leftover AFM matrix in a second step (e.g., by heating) [13–14]. The drawback of this approach is the lack of serious control over the size and density of the precipitates in the matrix. The alternative is to co-deposit preformed FM clusters (e.g., by inert gas-condensation) and AFM matrices [9–11]. In that case the cluster size can be well-defined and, having control over the exact deposition rates of the clusters and the matrix, the amount of clusters can also be exactly adjusted. However, to date, only a few studies on the EB in cluster/matrix systems have been published, most of them being based on a very limited number of samples.

In this paper, a rather comprehensive study of the magnetic characteristics in the system of preformed Fe clusters embedded in Cr matrices is presented. It is based on the largest series of samples (20) for any FM/AFM cluster/matrix combination reported in literature. Due to the large amount of samples representing three different cluster sizes and a broad range of cluster concentrations in the matrix, combined with a high degree of control over the experimental conditions, the effects of the two critical parameters, cluster size and density in the matrix on *T*_B_, *H*_c_ and *H*_eb_ could be clearly shown. The system Fe*_x_*/Cr is a perfect model system: being just based on two components (Cr is an AFM element), it avoids the pitfall of compositional variations in the AFM (e.g., only partially oxidized CoO) which may lead to additional, unwanted effects.

## Results and Discussion

The samples were prepared in a newly developed UHV cluster ion beam deposition apparatus, which is described elsewhere [2]. Fe clusters are produced in a Haberland-type magnetron sputtering/gas aggregation cluster source. Extracted anions are accelerated by electrostatic lenses and mass-separated in a 90° sector magnet. The mass resolution depends on the cluster size and can be estimated to be better than 1/10 for the utilized clusters. Prior to deposition the clusters are decelerated to 50 eV and then soft-landed on a silicon substrate with a native oxide layer (still conducting). To avoid migration and agglomeration of the clusters the substrate is cooled with liquid nitrogen during deposition. The Cr matrix is co-deposited by using an effusion cell. The flux of matrix material is monitored by a quartz crystal thickness monitor and the cluster flux by counting charges impinging on the sample area with a picoamperemeter (the clusters are singly charged). Counting the charges in combination with the known cluster mass from the mass separation the amount of deposited cluster material can be precisely derived. In order to minimize contamination with, e.g., oxygen the pressure in the deposition chamber is maintained in the 10^−9^ mbar range during the deposition.

Fe*_x_*/Cr samples consist of the already mentioned Si substrate with a native oxide layer, a 10 nm Cr base layer, the Fe cluster/Cr matrix layer, a 10 nm Cr top layer and a 10 nm Au film as oxidation protection. This geometry makes sure that the Fe clusters are in contact with Cr only and no oxidation takes place after deposition. To allow for a detailed comparative study of the magnetic characteristics of the samples the absolute amount of deposited Fe is the same for all samples, namely a 6 nm equivalent film thickness of clusters, and the Fe cluster concentration was adjusted by the amount of deposited Cr. Fe*_x_*/Cr samples were produced with Fe cluster sizes of 500, 1000 and 2000 atoms per cluster, corresponding to cluster diameters of 2.3, 2.8 and 3.6 nm, respectively and cluster volume fractions ranging from 2 to 50 vol %. For the three cluster sizes (500, 1000 and 2000 atoms/cluster) the aforementioned deposition energy of 50 eV results in 0.1, 0.05 and 0.025 eV/atom, respectively, which is clearly below the binding energy per atom. Therefore, fragmentation as well as pronounced deformation of the clusters during landing can be excluded [15].

**Figure 1:**
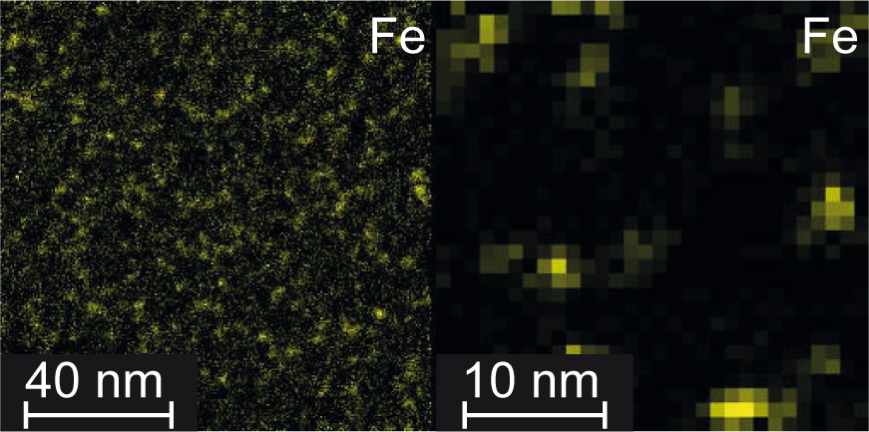
EFTEM (left) and STEM (right) micrographs of a 10 vol % Fe_1000_/Cr sample prepared on a TEM grid + amorphous carbon film with an Fe cluster equivalent thickness of 0.2 nm. The EFTEM image shows the Fe cluster distribution in the sample and the STEM image individual Fe clusters, it was recorded using EDX and the Fe K signal.

Figure 1 shows energy-filtered transmission electron microscopy (EFTEM) and scanning transmission electron microscopy (STEM) micrographs of the Fe distribution for a 10 vol % Fe_1000_/Cr sample, specifically prepared for TEM. To avoid subsequent focused ion beam cutting and possible oxidation, the sample was deposited on a TEM grid covered with a thin amorphous carbon film while the whole sample thickness including top and bottom Cr layers was just 5 nm. Deposition parameters such as the cluster deposition rate and the sample temperature during deposition were identical with the ones used for the other samples. The EFTEM micrograph clearly shows that the Fe clusters are evenly distributed in the matrix and no significant agglomeration occurs. In the STEM image individual Fe clusters are clearly visible. Their size can be estimated to be roughly 3 nm which matches the expected 2.8 nm. Additional diffraction data from TEM (not shown here) revealed that the Fe clusters as well as the Cr matrix both retain the bcc structure as expected.

In the following paragraphs the magnetic properties of the Fe cluster assemblies in the Cr matrix are discussed. The magnetic characteristics are extracted from standard zero-field cooled/field cooled (ZFC/FC) magnetization measurements and magnetic hysteresis loops recorded in a commercial superconducting quantum interference device (SQUID, Quantum Design) magnetometer.

The ZFC/FC curves were collected with an applied external magnetic field of μ_0_*H* = 20 mT in a temperature range between 10 and 350 K. The measurement geometry was in-plane, i.e., the external magnetic field was applied parallel to the sample surface (as for all magnetic data presented in this paper).

**Figure 2:**
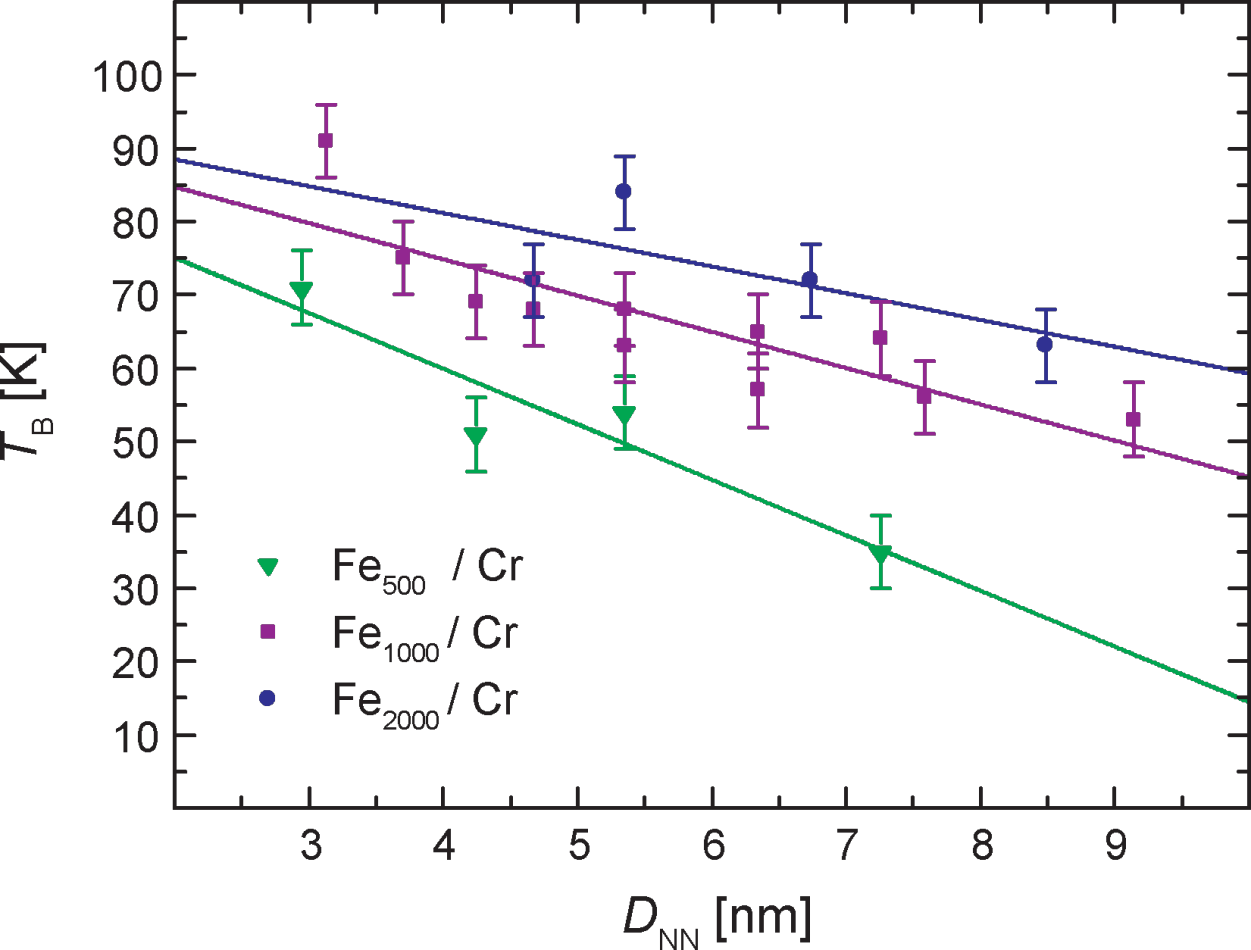
Blocking temperature *T*_B_ versus approximated nearest neighbor distances *D*_NN_ for Fe*_x_*/Cr samples. The solid lines on three series of samples with 500, 1000 and 2000 atoms are just guides to the eye. A clear dependence of *T*_B_ on the cluster size as well as *D*_NN_ is visible.

Figure 2 shows the *T*_B_ of the Fe*_x_*/Cr samples extracted from the ZFC/FC curves. At this point it is reasonable to assume that possible interactions between the clusters would not directly depend on the volume fraction of the clusters in the matrix, but on the average distances of neighboring clusters. To approximate this distance for the actually randomly distributed clusters, a body centered cubic (bcc) arrangement of clusters was assumed and the *T*_B_ (and subsequent magnetic data) were plotted versus the obtained nearest neighbor distances *D*_NN_. The data presented in this way reveal, that *T*_B_ is indeed affected both by the size of the embedded Fe clusters as well as *D*_NN_. The values of *T*_B_ are higher for larger clusters and rise nearly linear (in the investigated region) with decreasing values of *D*_NN_ with the linear slope being smaller for larger clusters. Thus, the differences in *T*_B_ between the three cluster sizes become distinctly smaller at smaller *D*_NN_ (higher volume fraction of the clusters). To minimize the influence of inter-cluster interactions the dependency on the cluster size should be first considered for the larger cluster distances. As a starting point for the analysis one could refer to the simplest model of non-interacting particles with an uniaxial anisotropy in a non-magnetic matrix. Here one would expect a simple proportionality 
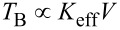
, where *K*_eff_ is an effective anisotropy constant and *V* the particle volume. Indeed, the measured *T*_B_ show some rudimentary size dependence, especially at large *D*_NN_, but they do not scale linearly with the cluster size. Also the estimated *K*_eff_ of (0.8–1.3) × 10^6^ J/m^3^ is almost two orders of magnitude bigger than one would expect for clusters with the magnetocrystalline anisotropy of bulk α-iron. Both results lead to the conclusion that for the lowest concentration of clusters the effective anisotropy constant is determined by magnetic exchange interactions with the Cr matrix, which is substantiated by a direct comparison with the Fe clusters embedded in a nonmagnetic Ag matrix. The Fe_1000_ clusters with *D*_NN_ ≈ 9 nm (2 vol % Fe) were studied earlier and their *T*_B_ was about 6 K [2]. An increase of the *T*_B_ by almost one order of magnitude to 53 K, for the Fe_1000_ clusters deposited in the AFM Cr matrix unambiguously points out the decisive role of FM/AFM exchange coupling in the enhancement of the magnetic anisotropy. For lower *D*_NN_ the effect of the particle size on *T*_B_ gets even less pronounced, since magnetic inter-particle interactions (e.g., strong dipole–dipole interactions) become dominant.

**Figure 3:**
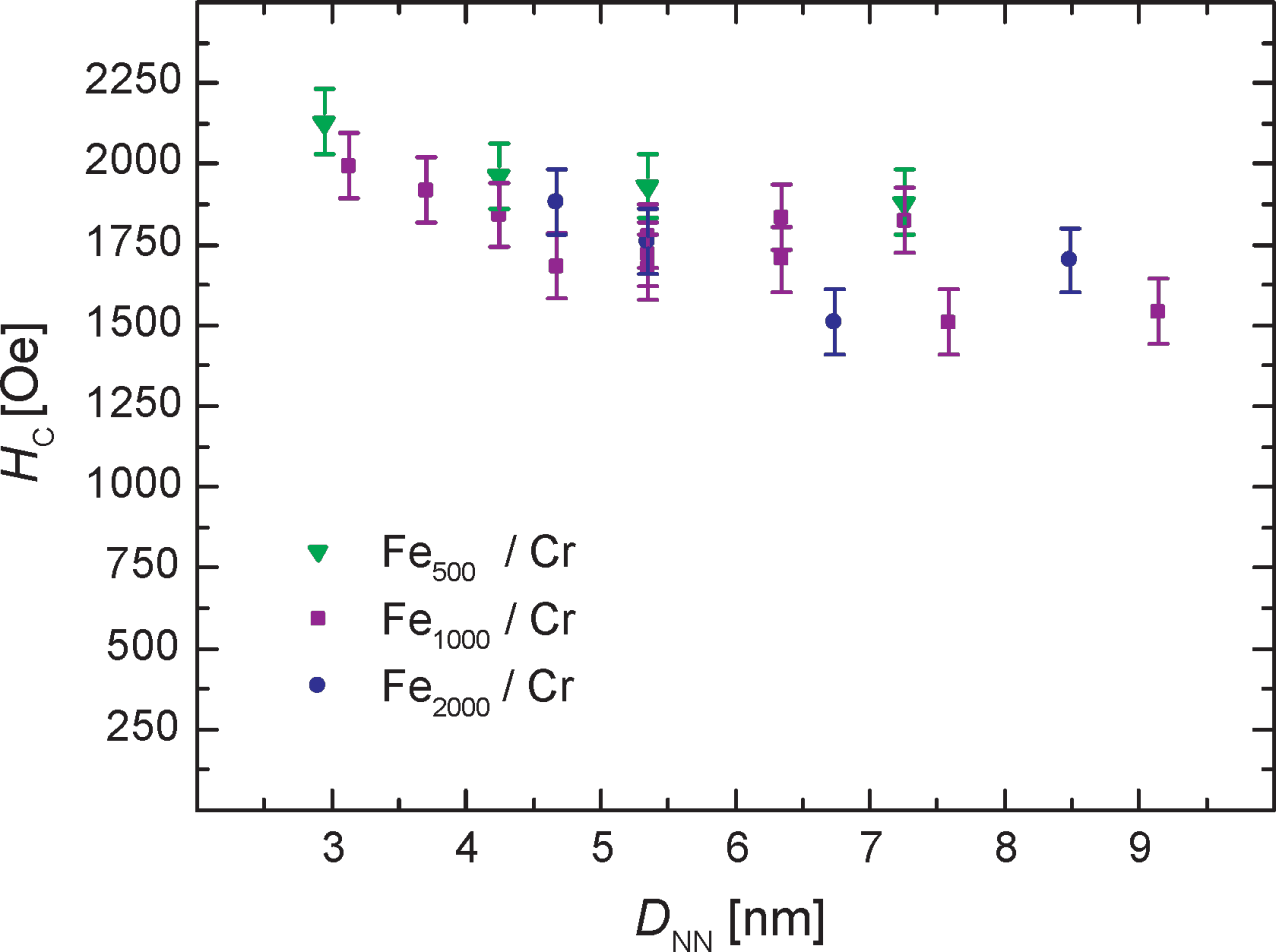
*H*_c_ versus *D*_NN_ for Fe*_x_*/Cr samples. *H*_c_ mainly depends on *D*_NN_, no clear effect of the cluster size is visible in the investigated region.

Hysteresis loops were recorded at 5 K after field cooling from 350 K, which is above the *T*_N_ of Cr (311 K [16]), in an external magnetic field of μ_0_*H* = 4.5 T. A linear diamagnetic background originating from the Si substrate as well as the Au layers was subtracted. The coercivity *H*_c_ of the samples can be derived from the recorded hysteresis loops as 
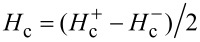
, with 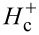 and 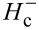 being the external field values μ_0_*H* for which the magnetization *M* = 0 at the positive and negative branches of the magnetic hysteresis loops respectively. The obtained values of *H*_c_ for the three sample series are shown in Figure 3.

In the investigated region *H*_c_ shows no clear dependence on the size of the embedded Fe clusters, but rises slightly with decreasing *D*_NN_ from roughly 1550 Oe (*D*_NN_ ≈ 9 nm) to around 2000 Oe (*D*_NN_ ≈ 3 nm). This behavior shows that *H*_c_ mainly depends on the local anisotropy of the Fe clusters and rather weakly rises for smaller *D*_NN_ due to extra anisotropy from interactions between the individual Fe clusters. Comparing again the Fe_1000_/Cr sample with *D*_NN_ ≈ 9 nm (2 vol % Fe) with the above mentioned Fe_1000_/Ag sample with the same cluster volume fraction a distinct rise in *H*_c_ from 56 Oe for Fe_1000_/Ag to 1543 Oe for Fe_1000_/Cr is found, underlining again the distinct change of the anisotropy constant *K*_eff_ due to the FM/AFM interactions with the Cr matrix.

**Figure 4:**
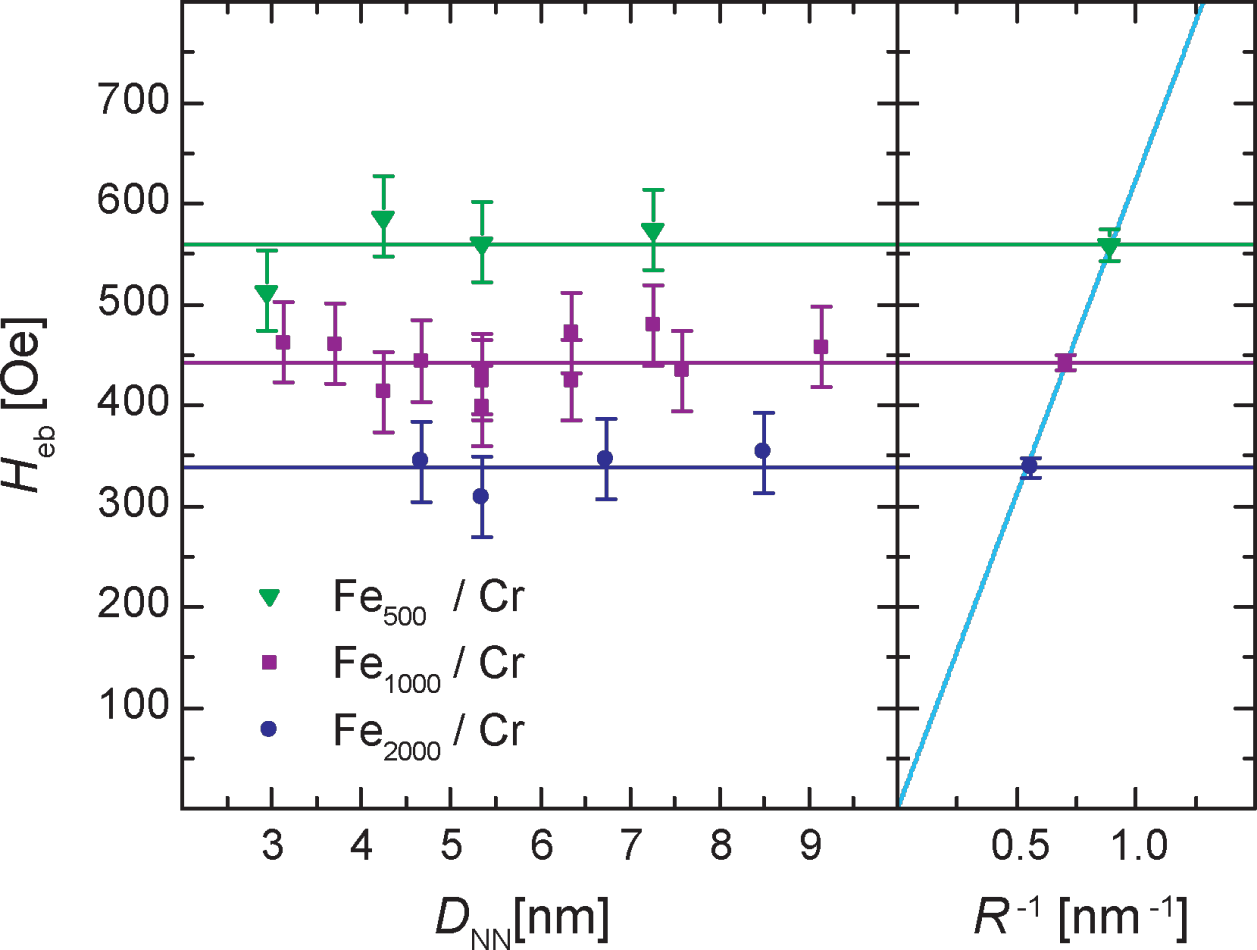
Left: *H*_eb_ versus *D*_NN_ for the three series of samples with different cluster sizes. *D*_NN_ has no effect on *H*_eb_, while a pronounced effect is found for the size of the embedded clusters. Right: Average *H*_eb_ versus *R*^−1^ for the three cluster sizes showing a linear relation.

The horizontal shift of the magnetic hysteresis loops is described by 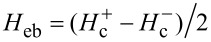. Figure 4 shows the values of *H*_eb_ extracted from the magnetic hysteresis loops of the different samples. The EB values are basically independent of *D*_NN_. The largest series of Fe_1000_/Cr samples exhibits almost linear behavior with a negligible slope of 2.4(4.5) nm·Oe^−1^. Therefore, the data can be described within the error with a horizontal line, implying that the volume fraction of the Fe clusters has either no or only little influence on *H*_eb_. On the other hand comparing the average *H*_eb_ values of the three series with different cluster sizes a pronounced effect of the cluster size on *H*_eb_ is clearly visible. Fitting horizontal lines to each series one obtains average values of *H*_eb_ of 559(16) Oe, 442(7) Oe and 338(10) Oe for Fe_500_/Cr, Fe_1000_/Cr and Fe_2000_/Cr, respectively.

To model the dependence of *H*_eb_ on the cluster size one should keep in mind that in first approximation the EB is an interface phenomenon. When the FM Fe clusters are cooled in the external magnetic field down below *T*_N_ of the Cr matrix the clusters lock AFM Cr domains in a certain direction via exchange FM/AFM interactions. The initial orientation of the AFM domains determines the unidirectional anisotropy axis resulting in the shift of the magnetization hysteresis loops. For spherical FM clusters one can assume that FM spins residing on the surface are exchange-coupled to the AFM neighbors with a strength determined by the exchange integral *J*. The FM/AFM interaction keeps all the spins of the cluster along the unidirectional anisotropy axis. During the magnetization switching process the external magnetic field flips the magnetization in the opposite direction. The switching field must overcome the FM/AFM coupling which is proportional to *J* multiplied by the cluster surface area π*R*^2^, where *R* is the cluster radius. On the other hand the bigger the total magnetic moment of the cluster (which is proportional to the number of magnetic moments per cluster and, thus, to the volume, i.e., to *R*^3^) the higher is the torque induced by the external magnetic field and the easier is the rotation away from an easy axis. Thus the switching field is proportional to the ratio *J*·*R*^2^/*R*^3^, which eventually results in 
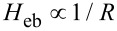
. Plotting the obtained *H*_eb_ values for the three cluster sizes versus 1/*R* of the clusters (Figure 4) and assuming *H*_eb_ = 0 for an infinitely large particle (1/*R* = 0) a linear relation is found in the investigated region of cluster radii and a linear fit to the data yields a slope of 624(7) Oe^−1^·nm^−1^. This straightforward relation between *H*_eb_ and *R* of the embedded clusters has never been shown to that degree in any FM/AFM cluster/matrix system.

As a comparison, one can look at the closely related core/shell nanoparticles featuring a FM core and an AFM shell. In that case a theoretical study predicted an oscillatory relation of *H*_eb_ and *R* [17]. On the other hand one can also refer to thin film systems composed of a FM and an AFM layer. It was shown by Restrepo-Parra et. al. [18] that 
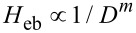
, with *m* ≈ 1 and *D* being the thickness of the FM layer. This result nicely supports our finding, since it became clear in both studies that *H*_eb_ is basically proportional to the surface to volume or interface to volume ratio of the FM part of the system.

Fe*_x_*/Cr was already studied in a previous publication by Qureshi et al. [11]. It is based on three samples with different volume fractions of clusters of a single size (≈340 atoms/cluster) and amongst others they report on *H*_c_ and *H*_eb_ that were both found to rise with rising volume fraction of the clusters. Compared to the results shown in the present study the behavior of *H*_c_ exhibits a similar trend with the absolute values being three to five times lower. For *H*_eb_ the values are between two and five times lower than the lowest *H*_eb_ observed here (310 Oe). In addition they show a dependence on the volume fraction of the clusters, which is not validated in the present study. Of course these discrepancies cannot be easily addressed, but it needs to be stated that it was found in first test experiments that a high degree of control over the deposition parameters is of utmost importance for the consistency of the obtained data. For instance, in trial experiments the sample temperature varied during the deposition from sample to sample due to different evaporator temperatures or erratic thermal contact of the sample to the sample holder which resulted in quite different magnetic characteristics. Only after cooling the samples with liquid nitrogen during deposition and gluing the samples to the sample holders with silver glue as well as keeping the deposition times similar for all samples it was possible to get reproducible and consistent results.

In conclusion, by using a dedicated UHV cluster-deposition apparatus we fabricated in a highly controllable way series of samples with Fe clusters embedded in Cr matrices. Subsequently, the magnetic characteristics of 20 samples with three different cluster sizes and varied cluster volume fractions were studied to determine their relevant parameters: *T*_B_, *H*_c_ and *H*_eb_. While *T*_B_ is found to be dependent on the size of the embedded clusters as well as on the average distance between neighboring clusters *D*_NN_, *H*_c_ is found to depend rather weakly on *D*_NN_. The exchange bias field *H*_eb_ responds to the size of the embedded clusters (
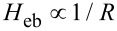
) but is actually not depending on the cluster concentration. With this observation one arrives at the conclusion that the exchange bias effect is a rather local effect limited to a few layers of the AFM Cr surrounding the FM Fe cluster.
